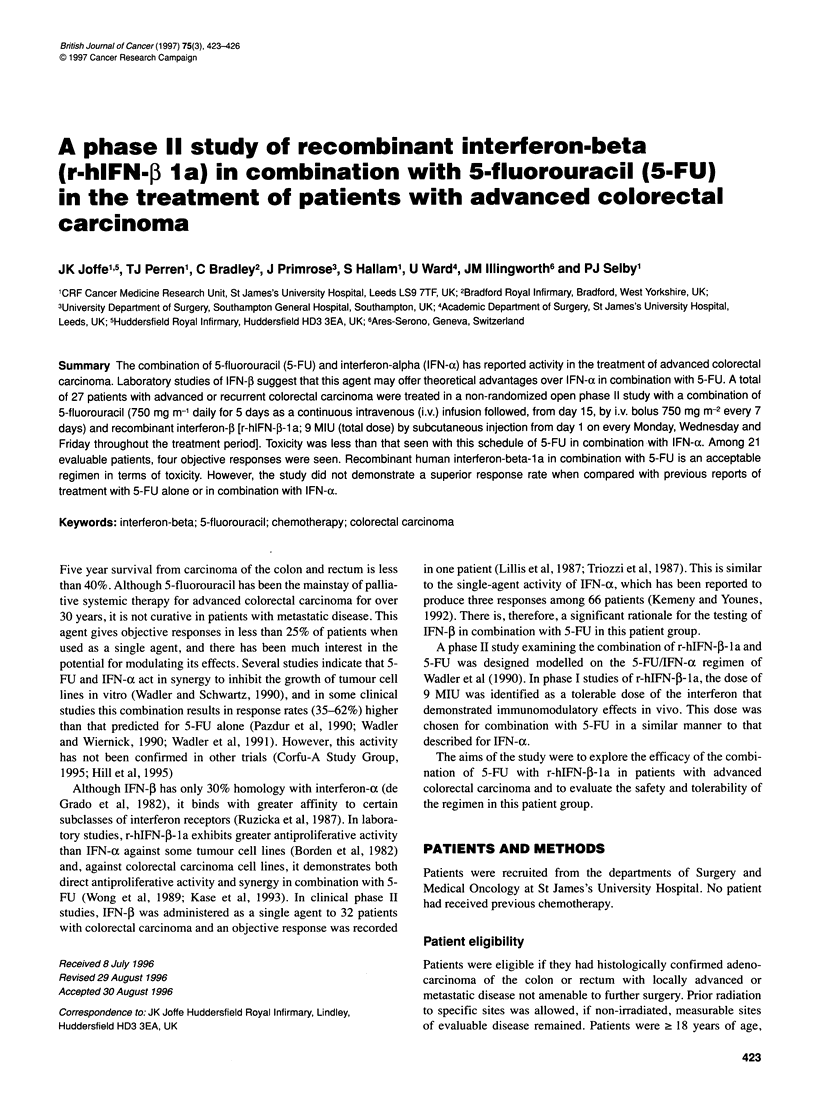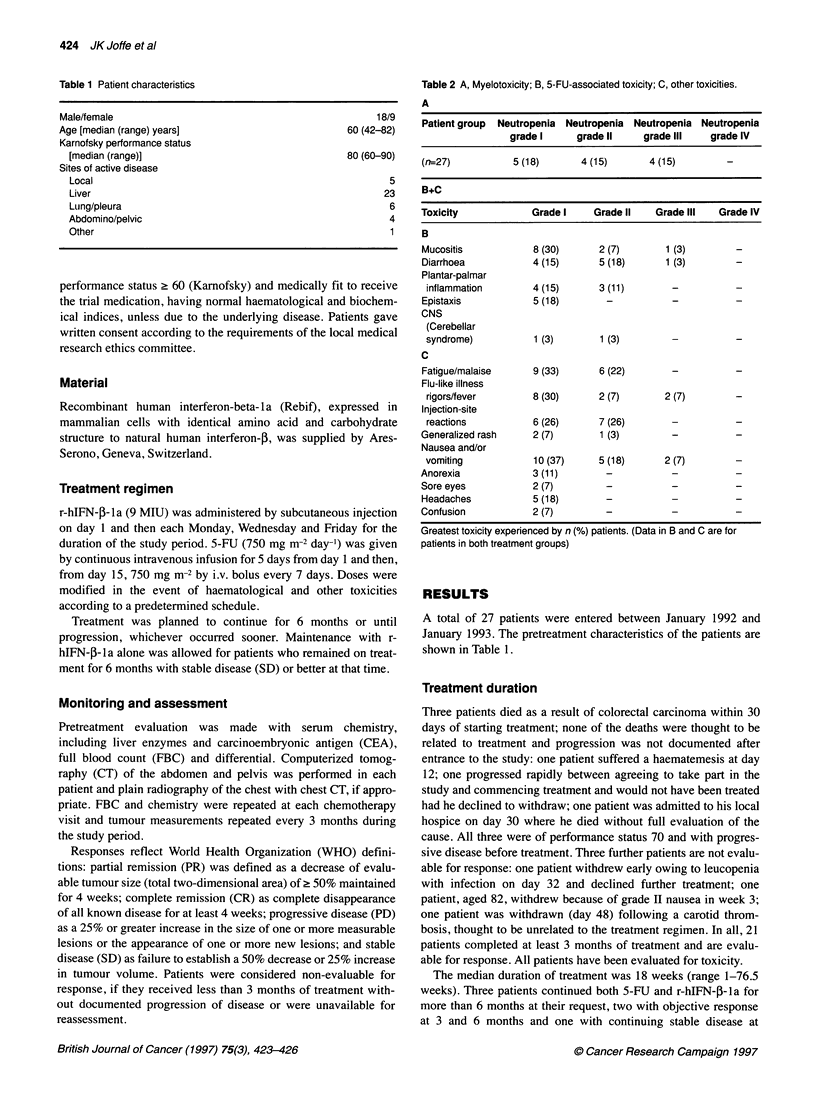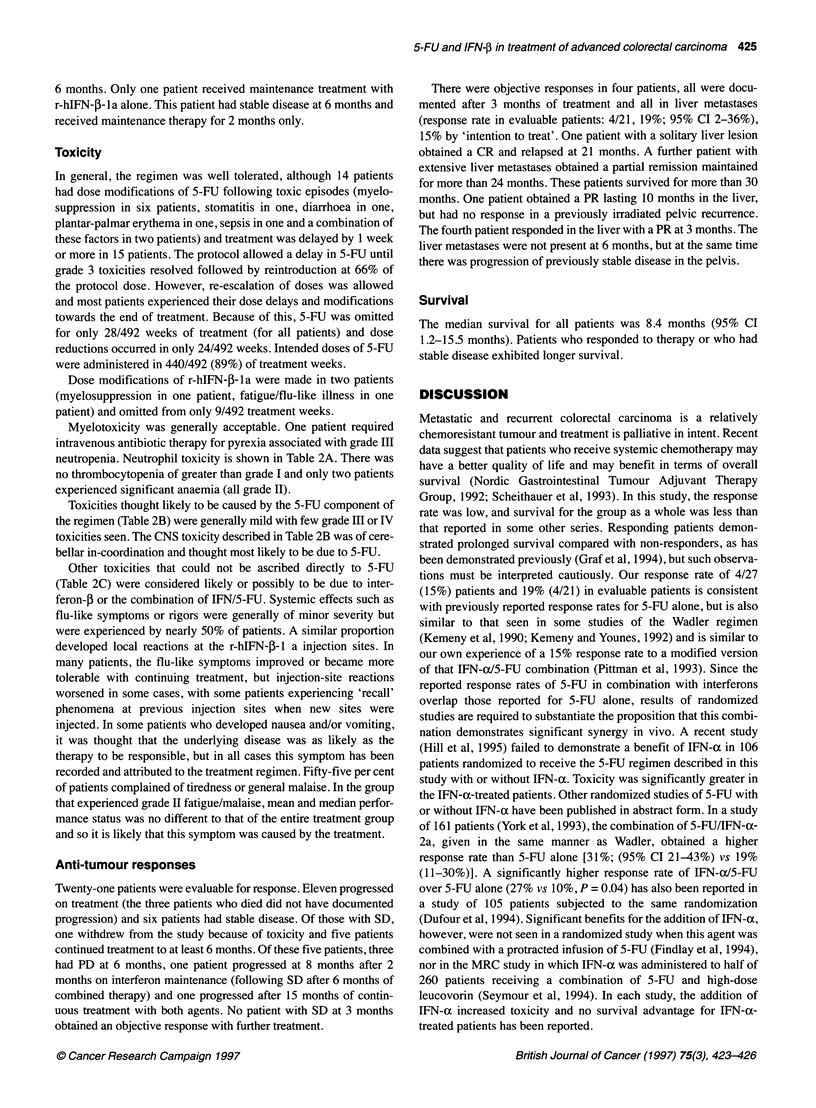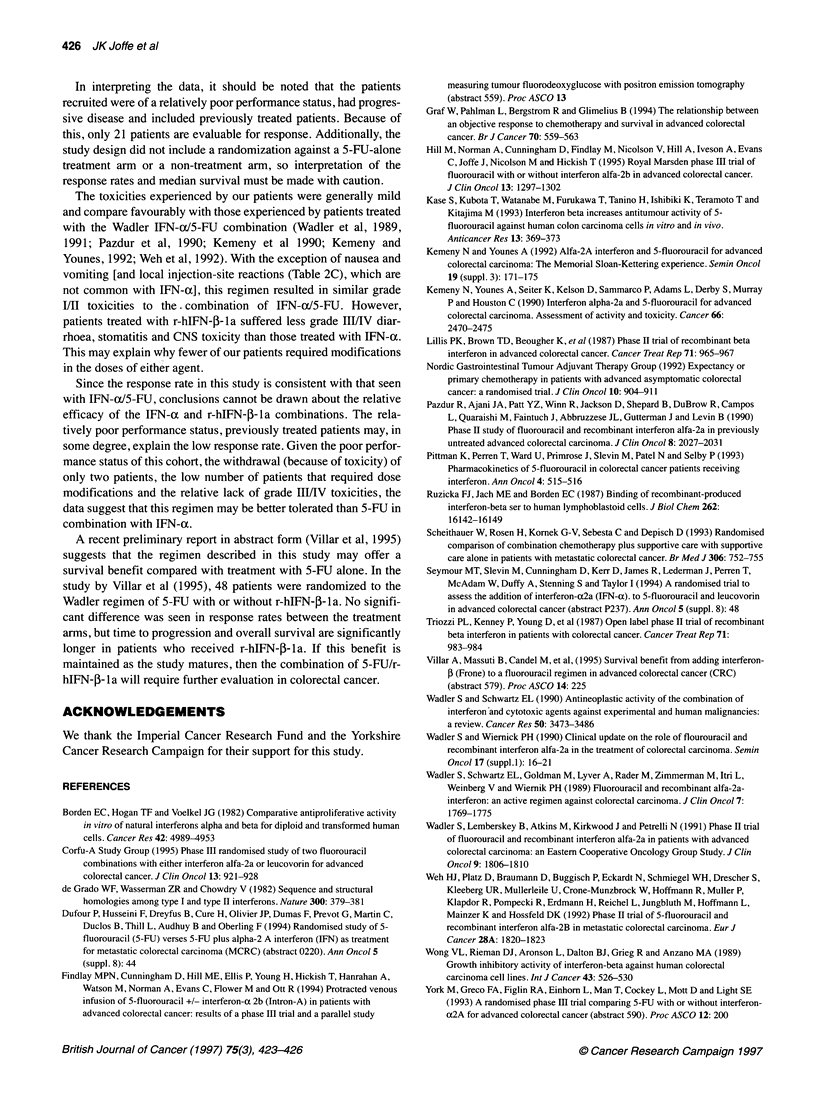# A phase II study of recombinant interferon-beta (r-hIFN-beta 1a) in combination with 5-fluorouracil (5-FU) in the treatment of patients with advanced colorectal carcinoma.

**DOI:** 10.1038/bjc.1997.69

**Published:** 1997

**Authors:** J. K. Joffe, T. J. Perren, C. Bradley, J. Primrose, S. Hallam, U. Ward, J. M. Illingworth, P. J. Selby

**Affiliations:** CRF Cancer Medicine Research Unit, St James's University Hospital, Leeds, UK.

## Abstract

The combination of 5-fluorouracil (5-FU) and interferon-alpha (IFN-alpha) has reported activity in the treatment of advanced colorectal carcinoma. Laboratory studies of IFN-beta suggest that this agent may offer theoretical advantages over IFN-alpha in combination with 5-FU. A total of 27 patients with advanced or recurrent colorectal carcinoma were treated in a non-randomized open phase II study with a combination of 5-fluorouracil (750 mg m(-1) daily for 5 days as a continuous intravenous (i.v.) infusion followed, from day 15, by i.v. bolus 750 mg m(-2) every 7 days) and recombinant interferon-beta [r-hIFN-beta-1a; 9 MIU (total dose) by subcutaneous injection from day 1 on every Monday, Wednesday and Friday throughout the treatment period]. Toxicity was less than that seen with this schedule of 5-FU in combination with IFN-alpha. Among 21 evaluable patients, four objective responses were seen. Recombinant human interferon-beta-1a in combination with 5-FU is an acceptable regimen in terms of toxicity. However, the study did not demonstrate a superior response rate when compared with previous reports of treatment with 5-FU alone or in combination with IFN-alpha.


British Journal of Cancer (1997) 75(3), 423–426
© 1997 Cancer Research Campaign

### A phase II study of recombinant interferon-beta (r-hIFN-β 1a) in combination with 5-fluorouracil (5-FU) in the treatment of patients with advanced colorectal carcinoma

JK Joffe[1,5], TJ Perren[1], C Bradley[2], J Primrose[3], S Hallam[1], U Ward[4], JM Illingworth[6] and PJ Selby[1]

[1]CRF Cancer Medicine Research Unit, St James's University Hospital, Leeds LS9 7TF, UK; [2]Bradford Royal Infirmary, Bradford, West Yorkshire, UK; [3]University Department of Surgery, Southampton General Hospital, Southampton, UK; [4]Academic Department of Surgery, St James's University Hospital, Leeds, UK; [5]Huddersfield Royal Infirmary, Huddersfield HD3 3EA, UK; [6]Ares-Serono, Geneva, Switzerland

**Summary** The combination of 5-fluorouracil (5-FU) and interferon-alpha (IFN-α) has reported activity in the treatment of advanced colorectal carcinoma. Laboratory studies of IFN-β suggest that this agent may offer theoretical advantages over IFN-α in combination with 5-FU. A total of 27 patients with advanced or recurrent colorectal carcinoma were treated in a non-randomized open phase II study with a combination of 5-fluorouracil (750 mg $m^{-1}$ daily for 5 days as a continuous intravenous (i.v.) infusion followed, from day 15, by i.v. bolus 750 mg $m^{-2}$ every 7 days) and recombinant interferon-β [r-hIFN-β-1a; 9 MIU (total dose) by subcutaneous injection from day 1 on every Monday, Wednesday and Friday throughout the treatment period]. Toxicity was less than that seen with this schedule of 5-FU in combination with IFN-α. Among 21 evaluable patients, four objective responses were seen. Recombinant human interferon-beta-1a in combination with 5-FU is an acceptable regimen in terms of toxicity. However, the study did not demonstrate a superior response rate when compared with previous reports of treatment with 5-FU alone or in combination with IFN-α.

**Keywords:** interferon-beta; 5-fluorouracil; chemotherapy; colorectal carcinoma

Five year survival from carcinoma of the colon and rectum is less than 40%. Although 5-fluorouracil has been the mainstay of palliative systemic therapy for advanced colorectal carcinoma for over 30 years, it is not curative in patients with metastatic disease. This agent gives objective responses in less than 25% of patients when used as a single agent, and there has been much interest in the potential for modulating its effects. Several studies indicate that 5-FU and IFN-α act in synergy to inhibit the growth of tumour cell lines in vitro (Wadler and Schwartz, 1990), and in some clinical studies this combination results in response rates (35–62%) higher than that predicted for 5-FU alone (Pazdur et al, 1990; Wadler and Wiernick, 1990; Wadler et al, 1991). However, this activity has not been confirmed in other trials (Corfu-A Study Group, 1995; Hill et al, 1995)

Although IFN-β has only 30% homology with interferon-α (de Grado et al, 1982), it binds with greater affinity to certain subclasses of interferon receptors (Ruzicka et al, 1987). In laboratory studies, r-hIFN-β-1a exhibits greater antiproliferative activity than IFN-α against some tumour cell lines (Borden et al, 1982) and, against colorectal carcinoma cell lines, it demonstrates both direct antiproliferative activity and synergy in combination with 5-FU (Wong et al, 1989; Kase et al, 1993). In clinical phase II studies, IFN-β was administered as a single agent to 32 patients with colorectal carcinoma and an objective response was recorded in one patient (Lillis et al, 1987; Triozzi et al, 1987). This is similar to the single-agent activity of IFN-α, which has been reported to produce three responses among 66 patients (Kemeny and Younes, 1992). There is, therefore, a significant rationale for the testing of IFN-β in combination with 5-FU in this patient group.

A phase II study examining the combination of r-hIFN-β-1a and 5-FU was designed modelled on the 5-FU/IFN-α regimen of Wadler et al (1990). In phase I studies of r-hIFN-β-1a, the dose of 9 MIU was identified as a tolerable dose of the interferon that demonstrated immunomodulatory effects in vivo. This dose was chosen for combination with 5-FU in a similar manner to that described for IFN-α.

The aims of the study were to explore the efficacy of the combination of 5-FU with r-hIFN-β-1a in patients with advanced colorectal carcinoma and to evaluate the safety and tolerability of the regimen in this patient group.

### PATIENTS AND METHODS

Patients were recruited from the departments of Surgery and Medical Oncology at St James's University Hospital. No patient had received previous chemotherapy.

### Patient eligibility

Patients were eligible if they had histologically confirmed adenocarcinoma of the colon or rectum with locally advanced or metastatic disease not amenable to further surgery. Prior radiation to specific sites was allowed, if non-irradiated, measurable sites of evaluable disease remained. Patients were ≥ 18 years of age,

*Received 8 July 1996*
*Revised 29 August 1996*
*Accepted 30 August 1996*

*Correspondence to:* JK Joffe Huddersfield Royal Infirmary, Lindley, Huddersfield HD3 3EA, UK

**Table 1** Patient characteristics

| | |
|---|---|
| Male/female | 18/9 |
| Age [median (range) years] | 60 (42–82) |
| Karnofsky performance status [median (range)] | 80 (60–90) |
| Sites of active disease | |
| Local | 5 |
| Liver | 23 |
| Lung/pleura | 6 |
| Abdomino/pelvic | 4 |
| Other | 1 |

performance status ≥ 60 (Karnofsky) and medically fit to receive the trial medication, having normal haematological and biochemical indices, unless due to the underlying disease. Patients gave written consent according to the requirements of the local medical research ethics committee.

### Material

Recombinant human interferon-beta-1a (Rebif), expressed in mammalian cells with identical amino acid and carbohydrate structure to natural human interferon-β, was supplied by Ares-Serono, Geneva, Switzerland.

### Treatment regimen r-hIFN-β-1a (9 MIU) was administered by subcutaneous injection on day 1 and then each Monday, Wednesday and Friday for the duration of the study period. 5-FU (750 mg $m^{-2}$ $day^{-1}$) was given by continuous intravenous infusion for 5 days from day 1 and then, from day 15, 750 mg $m^{-2}$ by i.v. bolus every 7 days. Doses were modified in the event of haematological and other toxicities according to a predetermined schedule.

Treatment was planned to continue for 6 months or until progression, whichever occurred sooner. Maintenance with r-hIFN-β-1a alone was allowed for patients who remained on treatment for 6 months with stable disease (SD) or better at that time.

### Monitoring and assessment

Pretreatment evaluation was made with serum chemistry, including liver enzymes and carcinoembryonic antigen (CEA), full blood count (FBC) and differential. Computerized tomography (CT) of the abdomen and pelvis was performed in each patient and plain radiography of the chest with chest CT, if appropriate. FBC and chemistry were repeated at each chemotherapy visit and tumour measurements repeated every 3 months during the study period.

Responses reflect World Health Organization (WHO) definitions: partial remission (PR) was defined as a decrease of evaluable tumour size (total two-dimensional area) of ≥ 50% maintained for 4 weeks; complete remission (CR) as complete disappearance of all known disease for at least 4 weeks; progressive disease (PD) as a 25% or greater increase in the size of one or more measurable lesions or the appearance of one or more new lesions; and stable disease (SD) as failure to establish a 50% decrease or 25% increase in tumour volume. Patients were considered non-evaluable for response, if they received less than 3 months of treatment without documented progression of disease or were unavailable for reassessment.

**Table 2** A, Myelotoxicity; B, 5-FU-associated toxicity; C, other toxicities.

**A**

| Patient group | Neutropenia grade I | Neutropenia grade II | Neutropenia grade III | Neutropenia grade IV |
|---|---|---|---|---|
| (*n*=27) | 5 (18) | 4 (15) | 4 (15) | – |

**B+C**

| Toxicity | Grade I | Grade II | Grade III | Grade IV |
|---|---|---|---|---|
| **B** | | | | |
| Mucositis | 8 (30) | 2 (7) | 1 (3) | – |
| Diarrhoea | 4 (15) | 5 (18) | 1 (3) | – |
| Plantar-palmar inflammation | 4 (15) | 3 (11) | – | – |
| Epistaxis | 5 (18) | – | – | – |
| CNS (Cerebellar syndrome) | 1 (3) | 1 (3) | – | – |
| **C** | | | | |
| Fatigue/malaise | 9 (33) | 6 (22) | – | – |
| Flu-like illness rigors/fever | 8 (30) | 2 (7) | 2 (7) | – |
| Injection-site reactions | 6 (26) | 7 (26) | – | – |
| Generalized rash | 2 (7) | 1 (3) | – | – |
| Nausea and/or vomiting | 10 (37) | 5 (18) | 2 (7) | – |
| Anorexia | 3 (11) | – | – | – |
| Sore eyes | 2 (7) | – | – | – |
| Headaches | 5 (18) | – | – | – |
| Confusion | 2 (7) | – | – | – |

Greatest toxicity experienced by *n* (%) patients. (Data in B and C are for patients in both treatment groups)

### RESULTS

A total of 27 patients were entered between January 1992 and January 1993. The pretreatment characteristics of the patients are shown in Table 1.

### Treatment duration

Three patients died as a result of colorectal carcinoma within 30 days of starting treatment; none of the deaths were thought to be related to treatment and progression was not documented after entrance to the study: one patient suffered a haematemesis at day 12; one progressed rapidly between agreeing to take part in the study and commencing treatment and would not have been treated had he declined to withdraw; one patient was admitted to his local hospice on day 30 where he died without full evaluation of the cause. All three were of performance status 70 and with progressive disease before treatment. Three further patients are not evaluable for response: one patient withdrew early owing to leucopenia with infection on day 32 and declined further treatment; one patient, aged 82, withdrew because of grade II nausea in week 3; one patient was withdrawn (day 48) following a carotid thrombosis, thought to be unrelated to the treatment regimen. In all, 21 patients completed at least 3 months of treatment and are evaluable for response. All patients have been evaluated for toxicity.

The median duration of treatment was 18 weeks (range 1–76.5 weeks). Three patients continued both 5-FU and r-hIFN-β-1a for more than 6 months at their request, two with objective response at 3 and 6 months and one with continuing stable disease at

© Cancer Research Campaign 1997

6 months. Only one patient received maintenance treatment with r-hIFN-β-1a alone. This patient had stable disease at 6 months and received maintenance therapy for 2 months only.

### Toxicity

In general, the regimen was well tolerated, although 14 patients had dose modifications of 5-FU following toxic episodes (myelosuppression in six patients, stomatitis in one, diarrhoea in one, plantar-palmar erythema in one, sepsis in one and a combination of these factors in two patients) and treatment was delayed by 1 week or more in 15 patients. The protocol allowed a delay in 5-FU until grade 3 toxicities resolved followed by reintroduction at 66% of the protocol dose. However, re-escalation of doses was allowed and most patients experienced their dose delays and modifications towards the end of treatment. Because of this, 5-FU was omitted for only 28/492 weeks of treatment (for all patients) and dose reductions occurred in only 24/492 weeks. Intended doses of 5-FU were administered in 440/492 (89%) of treatment weeks.

Dose modifications of r-hIFN-β-1a were made in two patients (myelosuppression in one patient, fatigue/flu-like illness in one patient) and omitted from only 9/492 treatment weeks.

Myelotoxicity was generally acceptable. One patient required intravenous antibiotic therapy for pyrexia associated with grade III neutropenia. Neutrophil toxicity is shown in Table 2A. There was no thrombocytopenia of greater than grade I and only two patients experienced significant anaemia (all grade II).

Toxicities thought likely to be caused by the 5-FU component of the regimen (Table 2B) were generally mild with few grade III or IV toxicities seen. The CNS toxicity described in Table 2B was of cerebellar in-coordination and thought most likely to be due to 5-FU.

Other toxicities that could not be ascribed directly to 5-FU (Table 2C) were considered likely or possibly to be due to interferon-β or the combination of IFN/5-FU. Systemic effects such as flu-like symptoms or rigors were generally of minor severity but were experienced by nearly 50% of patients. A similar proportion developed local reactions at the r-hIFN-β-1 a injection sites. In many patients, the flu-like symptoms improved or became more tolerable with continuing treatment, but injection-site reactions worsened in some cases, with some patients experiencing 'recall' phenomena at previous injection sites when new sites were injected. In some patients who developed nausea and/or vomiting, it was thought that the underlying disease was as likely as the therapy to be responsible, but in all cases this symptom has been recorded and attributed to the treatment regimen. Fifty-five per cent of patients complained of tiredness or general malaise. In the group that experienced grade II fatigue/malaise, mean and median performance status was no different to that of the entire treatment group and so it is likely that this symptom was caused by the treatment.

### Anti-tumour responses

Twenty-one patients were evaluable for response. Eleven progressed on treatment (the three patients who died did not have documented progression) and six patients had stable disease. Of those with SD, one withdrew from the study because of toxicity and five patients continued treatment to at least 6 months. Of these five patients, three had PD at 6 months, one patient progressed at 8 months after 2 months on interferon maintenance (following SD after 6 months of combined therapy) and one progressed after 15 months of continuous treatment with both agents. No patient with SD at 3 months obtained an objective response with further treatment.

There were objective responses in four patients, all were documented after 3 months of treatment and all in liver metastases (response rate in evaluable patients: 4/21, 19%; 95% CI 2–36%), 15% by 'intention to treat'. One patient with a solitary liver lesion obtained a CR and relapsed at 21 months. A further patient with extensive liver metastases obtained a partial remission maintained for more than 24 months. These patients survived for more than 30 months. One patient obtained a PR lasting 10 months in the liver, but had no response in a previously irradiated pelvic recurrence. The fourth patient responded in the liver with a PR at 3 months. The liver metastases were not present at 6 months, but at the same time there was progression of previously stable disease in the pelvis.

### Survival

The median survival for all patients was 8.4 months (95% CI 1.2–15.5 months). Patients who responded to therapy or who had stable disease exhibited longer survival.

### DISCUSSION

Metastatic and recurrent colorectal carcinoma is a relatively chemoresistant tumour and treatment is palliative in intent. Recent data suggest that patients who receive systemic chemotherapy may have a better quality of life and may benefit in terms of overall survival (Nordic Gastrointestinal Tumour Adjuvant Therapy Group, 1992; Scheithauer et al, 1993). In this study, the response rate was low, and survival for the group as a whole was less than that reported in some other series. Responding patients demonstrated prolonged survival compared with non-responders, as has been demonstrated previously (Graf et al, 1994), but such observations must be interpreted cautiously. Our response rate of 4/27 (15%) patients and 19% (4/21) in evaluable patients is consistent with previously reported response rates for 5-FU alone, but is also similar to that seen in some studies of the Wadler regimen (Kemeny et al, 1990; Kemeny and Younes, 1992) and is similar to our own experience of a 15% response rate to a modified version of that IFN-α/5-FU combination (Pittman et al, 1993). Since the reported response rates of 5-FU in combination with interferons overlap those reported for 5-FU alone, results of randomized studies are required to substantiate the proposition that this combination demonstrates significant synergy in vivo. A recent study (Hill et al, 1995) failed to demonstrate a benefit of IFN-α in 106 patients randomized to receive the 5-FU regimen described in this study with or without IFN-α. Toxicity was significantly greater in the IFN-α-treated patients. Other randomized studies of 5-FU with or without IFN-α have been published in abstract form. In a study of 161 patients (York et al, 1993), the combination of 5-FU/IFN-α-2a, given in the same manner as Wadler, obtained a higher response rate than 5-FU alone [31%; (95% CI 21–43%) *vs* 19% (11–30%)]. A significantly higher response rate of IFN-α/5-FU over 5-FU alone (27% *vs* 10%, $P = 0.04$) has also been reported in a study of 105 patients subjected to the same randomization (Dufour et al, 1994). Significant benefits for the addition of IFN-α, however, were not seen in a randomized study when this agent was combined with a protracted infusion of 5-FU (Findlay et al, 1994), nor in the MRC study in which IFN-α was administered to half of 260 patients receiving a combination of 5-FU and high-dose leucovorin (Seymour et al, 1994). In each study, the addition of IFN-α increased toxicity and no survival advantage for IFN-α-treated patients has been reported.

© Cancer Research Campaign 1997

In interpreting the data, it should be noted that the patients recruited were of a relatively poor performance status, had progressive disease and included previously treated patients. Because of this, only 21 patients are evaluable for response. Additionally, the study design did not include a randomization against a 5-FU-alone treatment arm or a non-treatment arm, so interpretation of the response rates and median survival must be made with caution.

The toxicities experienced by our patients were generally mild and compare favourably with those experienced by patients treated with the Wadler IFN-α/5-FU combination (Wadler et al, 1989, 1991; Pazdur et al, 1990; Kemeny et al 1990; Kemeny and Younes, 1992; Weh et al, 1992). With the exception of nausea and vomiting [and local injection-site reactions (Table 2C), which are not common with IFN-α], this regimen resulted in similar grade I/II toxicities to the combination of IFN-α/5-FU. However, patients treated with r-hIFN-β-1a suffered less grade III/IV diarrhoea, stomatitis and CNS toxicity than those treated with IFN-α. This may explain why fewer of our patients required modifications in the doses of either agent.

Since the response rate in this study is consistent with that seen with IFN-α/5-FU, conclusions cannot be drawn about the relative efficacy of the IFN-α and r-hIFN-β-1a combinations. The relatively poor performance status, previously treated patients may, in some degree, explain the low response rate. Given the poor performance status of this cohort, the withdrawal (because of toxicity) of only two patients, the low number of patients that required dose modifications and the relative lack of grade III/IV toxicities, the data suggest that this regimen may be better tolerated than 5-FU in combination with IFN-α.

A recent preliminary report in abstract form (Villar et al, 1995) suggests that the regimen described in this study may offer a survival benefit compared with treatment with 5-FU alone. In the study by Villar et al (1995), 48 patients were randomized to the Wadler regimen of 5-FU with or without r-hIFN-β-1a. No significant difference was seen in response rates between the treatment arms, but time to progression and overall survival are significantly longer in patients who received r-hIFN-β-1a. If this benefit is maintained as the study matures, then the combination of 5-FU/r-hIFN-β-1a will require further evaluation in colorectal cancer.

### ACKNOWLEDGEMENTS

We thank the Imperial Cancer Research Fund and the Yorkshire Cancer Research Campaign for their support for this study.

### REFERENCES

Borden EC, Hogan TF and Voelkel JG (1982) Comparative antiproliferative activity *in vitro* of natural interferons alpha and beta for diploid and transformed human cells. *Cancer Res* **42**: 4989–4953

Corfu-A Study Group (1995) Phase III randomised study of two fluorouracil combinations with either interferon alfa-2a or leucovorin for advanced colorectal cancer. *J Clin Oncol* **13**: 921–928

de Grado WF, Wasserman ZR and Chowdry V (1982) Sequence and structural homologies among type I and type II interferons. *Nature* **300**: 379–381

Dufour P, Husseini F, Dreyfus B, Cure H, Olivier JP, Dumas F, Prevot G, Martin C, Duclos B, Thill L, Audhuy B and Oberling F (1994) Randomised study of 5-fluorouracil (5-FU) verses 5-FU plus alpha-2 A interferon (IFN) as treatment for metastatic colorectal carcinoma (MCRC) (abstract 0220). *Ann Oncol* **5** (suppl. 8): 44

Findlay MPN, Cunningham D, Hill ME, Ellis P, Young H, Hickish T, Hanrahan A, Watson M, Norman A, Evans C, Flower M and Ott R (1994) Protracted venous infusion of 5-fluorouracil +/– interferon-α 2b (Intron-A) in patients with advanced colorectal cancer: results of a phase III trial and a parallel study measuring tumour fluorodeoxyglucose with positron emission tomography (abstract 559). *Proc ASCO* **13**

Graf W, Pahlman L, Bergstrom R and Glimelius B (1994) The relationship between an objective response to chemotherapy and survival in advanced colorectal cancer. *Br J Cancer* **70**: 559–563

Hill M, Norman A, Cunningham D, Findlay M, Nicolson V, Hill A, Iveson A, Evans C, Joffe J, Nicolson M and Hickish T (1995) Royal Marsden phase III trial of fluorouracil with or without interferon alfa-2b in advanced colorectal cancer. *J Clin Oncol* **13**: 1297–1302

Kase S, Kubota T, Watanabe M, Furukawa T, Tanino H, Ishibiki K, Teramoto T and Kitajima M (1993) Interferon beta increases antitumour activity of 5-fluorouracil against human colon carcinoma cells *in vitro* and *in vivo*. *Anticancer Res* **13**: 369–373

Kemeny N and Younes A (1992) Alfa-2A interferon and 5-fluorouracil for advanced colorectal carcinoma: The Memorial Sloan-Kettering experience. *Semin Oncol* **19** (suppl. 3): 171–175

Kemeny N, Younes A, Seiter K, Kelson D, Sammarco P, Adams L, Derby S, Murray P and Houston C (1990) Interferon alpha-2a and 5-fluorouracil for advanced colorectal carcinoma. Assessment of activity and toxicity. *Cancer* **66**: 2470–2475

Lillis PK, Brown TD, Beougher K, *et al* (1987) Phase II trial of recombinant beta interferon in advanced colorectal cancer. *Cancer Treat Rep* **71**: 965–967

Nordic Gastrointestinal Tumour Adjuvant Therapy Group (1992) Expectancy or primary chemotherapy in patients with advanced asymptomatic colorectal cancer: a randomised trial. *J Clin Oncol* **10**: 904–911

Pazdur R, Ajani JA, Patt YZ, Winn R, Jackson D, Shepard B, DuBrow R, Campos L, Quaraishi M, Faintuch J, Abbruzzese JL, Gutterman J and Levin B (1990) Phase II study of fluorouracil and recombinant interferon alfa-2a in previously untreated advanced colorectal carcinoma. *J Clin Oncol* **8**: 2027–2031

Pittman K, Perren T, Ward U, Primrose J, Slevin M, Patel N and Selby P (1993) Pharmacokinetics of 5-fluorouracil in colorectal cancer patients receiving interferon. *Ann Oncol* **4**: 515–516

Ruzicka FJ, Jach ME and Borden EC (1987) Binding of recombinant-produced interferon-beta ser to human lymphoblastoid cells. *J Biol Chem* **262**: 16142–16149

Scheithauer W, Rosen H, Kornek G-V, Sebesta C and Depisch D (1993) Randomised comparison of combination chemotherapy plus supportive care with supportive care alone in patients with metastatic colorectal cancer. *Br Med J* **306**: 752–755

Seymour MT, Slevin M, Cunningham D, Kerr D, James R, Lederman J, Perren T, McAdam W, Duffy A, Stenning S and Taylor I (1994) A randomised trial to assess the addition of interferon-α2a (IFN-α). to 5-fluorouracil and leucovorin in advanced colorectal cancer (abstract P237). *Ann Oncol* **5** (suppl. 8): 48

Triozzi PL, Kenney P, Young D, et al (1987) Open label phase II trial of recombinant beta interferon in patients with colorectal cancer. *Cancer Treat Rep* **71**: 983–984

Villar A, Massuti B, Candel M, et al, (1995) Survival benefit from adding interferon-β (Frone) to a fluorouracil regimen in advanced colorectal cancer (CRC) (abstract 579). *Proc ASCO* **14**: 225

Wadler S and Schwartz EL (1990) Antineoplastic activity of the combination of interferon and cytotoxic agents against experimental and human malignancies: a review. *Cancer Res* **50**: 3473–3486

Wadler S and Wiernick PH (1990) Clinical update on the role of flourouracil and recombinant interferon alfa-2a in the treatment of colorectal carcinoma. *Semin Oncol* **17** (suppl.1): 16–21

Wadler S, Schwartz EL, Goldman M, Lyver A, Rader M, Zimmerman M, Itri L, Weinberg V and Wiernik PH (1989) Fluorouracil and recombinant alfa-2a-interferon: an active regimen against colorectal carcinoma. *J Clin Oncol* **7**: 1769–1775

Wadler S, Lemberskey B, Atkins M, Kirkwood J and Petrelli N (1991) Phase II trial of fluorouracil and recombinant interferon alfa-2a in patients with advanced colorectal carcinoma: an Eastern Cooperative Oncology Group Study. *J Clin Oncol* **9**: 1806–1810

Weh HJ, Platz D, Braumann D, Buggisch P, Eckardt N, Schmiegel WH, Drescher S, Kleeberg UR, Mullerleile U, Crone-Munzbrock W, Hoffmann R, Muller P, Klapdor R, Pompecki R, Erdmann H, Reichel L, Jungbluth M, Hoffmann L, Mainzer K and Hossfeld DK (1992) Phase II trial of 5-fluorouracil and recombinant interferon alfa-2B in metastatic colorectal carcinoma. *Eur J Cancer* **28A**: 1820–1823

Wong VL, Rieman DJ, Aronson L, Dalton BJ, Grieg R and Anzano MA (1989) Growth inhibitory activity of interferon-beta against human colorectal carcinoma cell lines. *Int J Cancer* **43**: 526–530

York M, Greco FA, Figlin RA, Einhorn L, Man T, Cockey L, Mott D and Light SE (1993) A randomised phase III trial comparing 5-FU with or without interferon-α2A for advanced colorectal cancer (abstract 590). *Proc ASCO* **12**: 200

© Cancer Research Campaign 1997